# Abnormal left prefrontal N100 and its relationship with fronto-limbic metabolism in major depressive disorder

**DOI:** 10.1038/s41398-026-04107-1

**Published:** 2026-06-03

**Authors:** Shin-Wei Lin, Yuh-Feng Wang, Hui-Ching Lin, Chi-Hung Juan, Bang-Hung Yang, Reza Romorodi, Yoshihiro Noda, Chih-Ming Cheng, Jia-Shyun Jeng, Ya-Mei Bai, Cheng-Ta Li

**Affiliations:** 1 Precision Depression Intervention Center (PreDIC), Department of Psychiatry, Taipei Veterans General Hospital, Taipei, Taiwan; 2 Department of Psychiatry, Faculty of Medicine, National Yang Ming Chiao Tung University, Taipei, Taiwan; 3 Department of Nuclear Medicine, Taipei Veterans General Hospital, Taipei, Taiwan; 4 Department of Medical Imaging and Radiological Technology, Yuanpei University of Medical Technology, Hsinchu, Taiwan; 5 Department of Biomedical Imaging and Radiological Sciences, School of Biomedical Science and Engineering, National Yang Ming Chiao Tung University, Taipei, Taiwan; 6 Institute of Physiology, National Yang Ming Chiao Tung University, Taipei, Taiwan; 7 Institute of Cognitive Neuroscience, National Central University, Jhongli, Taiwan; 8 Temerty Centre for Therapeutic Brain Intervention, Centre for Addiction and Mental Health, Toronto, Ontario, Canada; 9 Department of Psychiatry, International University of Health and Welfare, Mita Hospital, Tokyo, 108-8329 Japan

**Keywords:** Molecular neuroscience, Depression

## Abstract

Dysfunction within inhibitory GABAergic systems is implicated in the pathophysiology of major depressive disorder (MDD). The N100, a transcranial magnetic stimulation-evoked potential (TEP), reflects inhibitory neural processes mediated by GABA-B receptors. Yet, the relationship between the N100, stress levels, and cerebral glucose metabolism in MDD remains under-investigated. We sought to elucidate the fundamental processes of frontal inhibitory function by examining the left prefrontal N100 in patients with MDD and healthy controls (HCs). Sixty-six patients with MDD and 20 HCs were recruited; the N100 component was derived from single-pulse TEPs targeting the left dorsolateral prefrontal cortex. Patients were stratified into low-severity (score ≤ 16) and high-severity (score ≥17) groups based on the 17-item Hamilton Depression Rating Scale (HDRS-17). Cerebral metabolic activity was estimated using 18F-FDG PET standardized uptake values (SUVs). N100 amplitudes were significantly greater in the low-severity depression group compared to HCs (p = 0.002) and exhibited a positive correlation with daily life stress exclusively in HCs (r = 0.653, p = 0.002). In the MDD group, higher N100 amplitudes were associated with increased SUVs in the prefrontal cortex (p = 0.037) and inversely correlated with SUVs in the right middle temporal cortex, right inferior parietal cortex, and middle cingulum (SVC-corrected p < 0.05). Furthermore, N100 amplitudes correlated positively with the number of treatment failures specifically in the non-treatment-resistant depression (nTRD) subgroup. These data indicate that the N100 is significantly associated with depression severity and may represent a dynamic, compensatory neurophysiological mechanism aiming to restore the excitatory/inhibitory balance in response to aberrant fronto-limbic metabolism.

## Introduction

Major depressive disorder (MDD) is a highly prevalent and disabling psychiatric illness affecting approximately 5% of the world’s population. It is expected to be the leading cause of disability by 2030 [1]. Despite the increased availability of treatment options in recent decades, treatment-resistant depression (TRD) patients who rarely respond to currently available antidepressant regimens continue to account for a significant proportion [2]. This underscores the importance of gaining a precise understanding of the pathophysiology of MDD.

Neuroimaging studies have reported variations in brain structure and functional connectivity among patients with MDD [3], and revealed potential mechanisms contributing to abnormalities in cognitive processing [4], mood regulation [5], impulsive behavior [6], and motivational function [7]. The inhibitory GABAergic systems are thought to play a crucial role in MDD pathogenesis, as evidenced by the promising antidepressant effects of ketamine [8]. These systems are also considered one of the key underlying mechanisms of Theta-burst stimulation (TBS) treatment [9]. TMS-evoked potentials (TEPs), assessed by concurrent transcranial magnetic stimulation and electroencephalography (TMS-EEG), are significantly associated with GABA receptor activity and may be consistently quantified from the prefrontal cortex (PFC) [10]. Furthermore, hypoactivity of the PFC, a crucial component of the fronto-limbic network, has been identified in MDD patients, and both treatment refractoriness and severity of symptoms were connected with the degree of PFC dysfunction [11, 12].

N100, a substantial negative deflection around 100 ms following a TMS pulse, is one of the most commonly studied TMS-EEG markers. It mostly represents inhibitory neuronal processes mediated by GABA-B receptors [13]. Several studies have reported that N100 is a possible predictor of antidepressant response and suicidal ideation in treatment-resistant patients, as well as being associated with symptom severity [14, 15]. Additionally, N100 has a high test-retest reliability and is less affected by signal decay and muscle artifacts [16, 17]. Collectively, these findings support the utility of the N100 as a promising biomarker for neurophysiological research in MDD.

The specific molecular mechanisms of the fronto-limbic circuit, which is associated with mood regulation, have yet to be fully determined. By integrating 18F-FDG PET imaging techniques, we sought to investigate brain region and circuit abnormalities from a whole-brain perspective, based on the established link between N100 and GABA-B receptor mediated inhibitory neuronal processes. Furthermore, a healthy control (HC) group was recruited to provide a comparative baseline. We expected that GABA-B receptor-mediated activity, as indexed by the N100 waveform, would correlate with depression severity and show significant associations with mood-regulating circuits and related brain regions.

## Methods

### Subjects

86 participants were included in this study, with 20 HC subjects and 66 MDD patients (low depression patients (N = 44) and high depression (N = 22)). A priori power calculation was performed to ensure a statistical power of at least 0.8, based on previous studies. All participants were aged from 20 to 70 years, and MDD patients fulfilled *the Diagnostic and Statistical Manual of Mental Disorders, Fourth Edition (DSM-IV)* criteria; the diagnosis was confirmed by thoroughly reviewing their medical history and conducting a semi-structured interview using the Mini International Neuropsychiatric Interview (MINI). No contraindications for TMS were found among patients. Participants were excluded if they had a lifetime history of psychiatric conditions such as psychotic disorders, bipolar disorders, or organic mental disorders, or significant medical or neurological conditions (e.g., traumatic brain injury, prior brain surgery, stroke, or seizures), were pregnant women, or had brain implants or cardiac pacemakers. Patients receiving more than 2 mg/day of lorazepam or any anticonvulsants were also excluded to avoid disrupting rTMS-EEG data collection. Informed written consent was collected from all participants, and this study was carried out in accordance with the ethical principles outlined in the Declaration of Helsinki. The study protocol was formally approved by the Institutional Review Board of Taipei Veterans General Hospital (Approval No. 2022-01-002B).

### Study overview and assessments

For all participants, information including treatment course, determined by the number of successful and unsuccessful antidepressant treatments, the total number of major depressive episodes, year of sickness, and depressive symptoms assessment using the 17-item Hamilton Depression Rating Scale (HDRS-17) [18], the Montgomery-Åsberg Depression Rating Scale (MADRS) [19], clinical global impression-severity (CGI) [20], and the Maudsley Staging Method (MSM) [21], was obtained. Regarding medication status at enrollment and during the study, 16.67% of the MDD patients were completely unmedicated. The remaining patients were receiving antidepressant monotherapy, which was recorded categorically as selective serotonin reuptake inhibitors (SSRIs), serotonin-norepinephrine reuptake inhibitors (SNRIs), tricyclic antidepressants (TCAs), or other atypical antidepressants. Specific daily dosages and exact durations of the current medication trials were not recorded. Standardized uptake value (SUV) proceeded from 18F-FDG PET/MR imaging and demonstrated the metabolic activity. TMS-evoked potential from the left dorsolateral prefrontal cortex was recorded from participants in both the HC group and the MDD group, and the N100 waveform was specifically extracted and evaluated.

### TMS-EEG experiments

We used a 70-mm figure-of-eight coil connected to two Magstim 200 stimulators via a Bi-Stim module (Magstim Company Ltd., Whitland, United Kingdom) to perform our TMS-EEG experiments. F3 was selected as the stimulation site due to its minimal inter-subject variability of recorded EEG signals. Stimulation intensity was set to 120% of the resting motor threshold (MT), which was determined from the area of the motor cortex that evoked the largest response in the right first dorsal interosseous muscle.

Fifty single and 50 paired pulses, which were given with a 100 ms interstimulus interval, were delivered to each subject randomly every 5 seconds. During TMS pulses, to downsize the acoustic effects, participants were asked to wear earplugs and then sit quietly with eyes open, looking straight ahead. By using a TMS-compatible 32-channel anti-CHamp EEG system (Brain Products GmbH, Gilching, Germany), EEG signals were recorded concomitantly under direct current (DC) mode with a low-pass filter at 100 Hz and a sampling rate of 20 kHz in order to record TMS-evoked potentials (TEP). We reported single-pulse TEP results in the present study. All scalp EEG electrode impedances were referenced to an electrode situated anterior to the Cz electrode and retained below 5 kΩ. To monitor and correct artifacts derived from eye movement, another four electrodes were placed around the eyes at the outer corners and above and below the left eye. To sum up, these measurement parameters were settled on to prevent amplifier saturation and to minimize TMS-related artifacts.

### TMS-EEG analysis

EEGLAB, TMSEEG (v5.0), and custom MATLAB scripts (R2018, The MathWorks, USA) following protocols established in previous articles [22, 23] were applied to process EEG data. Data was then epoched between −1000 and 1000 ms around the TMS pulses, with additional signal removal at 5 to 15 ms, baseline correction at −500 to −200 ms, removal of poor-quality channels, trials, and artifacts, and was filtered. We used independent component analysis (ICA) to further eliminate artifacts [23]. For the TMS marker single pulse N100, average data were calculated from the negative peak closest to 100 ms (80–120 ms) [22]. The detailed procedures were carried out largely as described previously [24]. The stimulation site was targeted at F3 for the study of left DLPFC, which plays a major role in MDD. In summary, we mainly followed the standard 10-step preprocessing pipeline as outlined previously, using the TMSEEG toolbox. This included filtering, ICA-based artifact rejection, and baseline correction procedures, which are widely accepted for mitigating decay artifacts. In the study, over 98% of subjects had no bad channels. A very small number of channels with obvious artifacts were removed from <2% of subjects. Trials with high amplitude artifacts (i.e., >1000 uV) were visually inspected and excluded when necessary.

### PET data acquisition and analysis

Participants were requested to fast for at least 8 h before receiving fluorodeoxyglucose (18F-FDG) PET scans to evaluate resting-state glucose metabolism of the brain through an integrated PET/MRI scanner (SIGNA; GE Healthcare, USA). After intravenous injection of approximately 370 MBq of 18F-FDG per 70 kg of body weight, scanning was performed 45 min afterwards in a dimly lit room while keeping participants awake [25]. 3D PET images acquired between 45 and 60 min post-injection were averaged and reconstructed using time-of-flight mode data with a 256 × 256 matrix, which used a 3D ordered subset expectation maximization (OSEM) algorithm set to 5 iterations, 32 subsets, and a 3-mm Gaussian filter.

Under a high-resolution sequence (repetition time: 2530 ms; echo spacing: 7.25 ms; echo time: 3 ms; flip angle: 7°), structural T1-weighted MRI images were recorded to deliver 1-mm isotropic voxels within a 256 × 256 mm field of view. Additionally, corrections for scatter, MRI-based attenuation, point spread function, dead time, and decay were performed. The PET data were analyzed using Statistical Parametric Mapping software (SPM12; Wellcome Department of Cognitive Neurology, University College London) within the MATLAB platform (version 7.1; The MathWorks Inc., Sherborn, MA, USA).

A specific 18F-FDG PET template was generated based on group MRI data [26, 27] for normalizing individual PET images. Through dividing the radiotracer activity concentration in the normalized PET image (MBq/kg) by the radiotracer dose (MBq) with body weight (kg) adjustment, parametric SUV images were calculated and then smoothed by conducting a Gaussian kernel with an 8-mm full-width at half maximum (FWHM) [25]. Voxel-wise partial correlation analyses were applied to clarify the relationship between TMS-EEG markers and the processed SUV images, and differences in brain SUVs between HC and MDD groups were also examined. Variables including age, gender, HDRS-17 scores, and global uptake levels were controlled [25]. We set the significance threshold at p < 0.05 at the voxel level, with family-wise error (FWE) correction for multiple comparisons. If the results were unable to meet the threshold, potential correlations were reported using an uncorrected voxel-level p < 0.005 and small volume correction (SVC) at p < 0.05 for exploratory purposes.

### Statistics methods

All statistical tests were two-sided. Data normality and homogeneity of variances were checked. One-way analysis of variance (ANOVA) and independent t-tests were applied to compare continuous variables between groups, inclusive of age, education, MSM, and HDRS-17, across three groups and two groups, respectively. When ANOVA revealed statistical significance (p-value < 0.05), a post-hoc analysis using the least significant difference (LSD) test was performed. The high depression group comprised patients with an HDRS-17 score of 17 or greater, while patients with a score of 16 or lower were classified as the low depression group. Fisher’s exact test and/or Yates’s correction were used for assessment of differences in categorical variables, such as gender. A two-way group-by-time ANOVA was performed to evaluate changes in TMS-EEG markers between groups, followed by a post-hoc analysis by LSD. A p-value of less than 0.05 was considered statistically significant. SPSS 21.0 (IBM, Armonk, NY, USA) was used for processing all statistical analyses, except for PET data.

## Results

There were no significant differences between the MDD and HC groups in demographic variables, including age, sex distribution, education level, or life stress scores. Significant differences were found in psychiatric hospitalizations (p = 0.013) and clinical scores, including MSM, HDRS-17, MADRS, and CGI rating scales. Notably, 15.2% of MDD patients experienced one or more episodes, and the significantly higher MSM scores (6.48 ± 2.35, p < 0.01) in MDD patients compared to the HC reflected illness severity and treatment refractoriness (Table 1). When comparing the three groups, including the HC, the low depression (N = 44), and the high depression (N = 22), significant differences were also found in psychiatric hospitalizations, MSM, HDRS-17, MADRS, and CGI scores (Table 2). Post-hoc analysis by LSD showed statistical significance between all pairs of groups in MSM, HDRS-17, MADRS, and CGI scores. For psychiatric hospitalizations, significant differences were found between the HC and high depression group, and between the low and high depression groups. To address potential confounding effects of gender on cortical inhibition, independent t-tests were conducted to examine sex differences in N100 amplitudes. No significant differences in N100 amplitudes were found between male and female participants across the entire study population (p = 0.559). Furthermore, no significant gender differences were observed when analyzing the HC (p = 0.184) and MDD (p = 0.520) groups independently.

**Table 1:** Demographic data and clinical variables of healthy controls (HCs) and major depressive disorder patients (MDD).

Variables	HC (N = 20)	MDD (N = 66)	p-value
Age (SD)	44.3 (16.6)	46.3 (14.6)	0.608
Sex			0.138
Male (%)	10(50.0)	21(31.8)	
Female (%)	10(50.0)	45(68.2)	
Education Level (%)			0.333
Elementary school	0(0)	2(3.0)	
Junior high school	0(0)	6(9.1)	
Senior high school	3(15.0)	14(21.2)	
College degree or higher	11(55.0)	34(51.5)	
Graduate school or higher	6(30.0)	10(15.2)	
No psychiatric hospitalization history (%)	20(100)	56(84.8)	0.013*
MSM (SD)	0(0)	6.48(2.35)	<0.001**
HDRS-17 (SD)	1.70(2.22)	10.40(8.32)	<0.001**
MADRS (SD)	2.10(3.29)	15.34(13.12)	<0.001**
Life stress total score (SD)	85.05(75.44)	100.67(99.57)	0.754
Clinical global impression (CGI) rating scales	0.95(0.22)	3.14(1.37)	<0.001**

**p < 0.01.

*p < 0.05.

**Table 2:** Demographic data and clinical variables across healthy controls (HCs), the high depression group and the low depression group.

Variables	HC (N = 20)	Low Depression (N = 44)	High Depression (N = 22)	p-value
Age (SD)	44.3(16.6)	47.5(13.6)	44.0(16.6)	0.594
Sex				0.088
Male (%)	10(50.0)	17(38.6)	4(18.2)	
Female (%)	10(50.0)	27(61.4)	18(81.8)	
Education Level (%)				0.336
Elementary school	0(0)	1(2.3)	1(4.5)	
Junior high school	0(0)	2(4.5)	4(18.2)	
Senior high school	3(15.0)	9(20.5)	5(22.7)	
College degree or higher	11(55.0)	24(54.5)	10(45.5)	
Graduate school or higher	6(30.0)	8(18.2)	2(9.1)	
No psychiatric hospitalization history (%)	20(100)	40(90.9)	16(72.7)	0.008*
MSM (SD)	0(0)	5.45(1.92)	8.55(1.71)	<0.001**
HDRS-17 (SD)	1.70(2.22)	5.27(3.77)	20.68(4.43)	<0.001**
MADRS (SD)	2.10(3.29)	7.93(7.35)	30.18(8.76)	<0.001**
Life stress total score (SD)	85.05(75.44)	98.45(84.22)	105.09(127.01)	0.785
CGI rating scales	0.95(0.22)	2.39(0.78)	4.64(1.00)	<0.001**

**p < 0.01.

*p < 0.05.

To assess the potential confounding effect of current antidepressant use on our neurophysiological markers, an ANOVA test was conducted comparing N100 amplitudes across the different medication status groups. This analysis revealed no significant differences in N100 amplitudes among these groups (F = 1.143, p = 0.348).

**Fig. 1:**
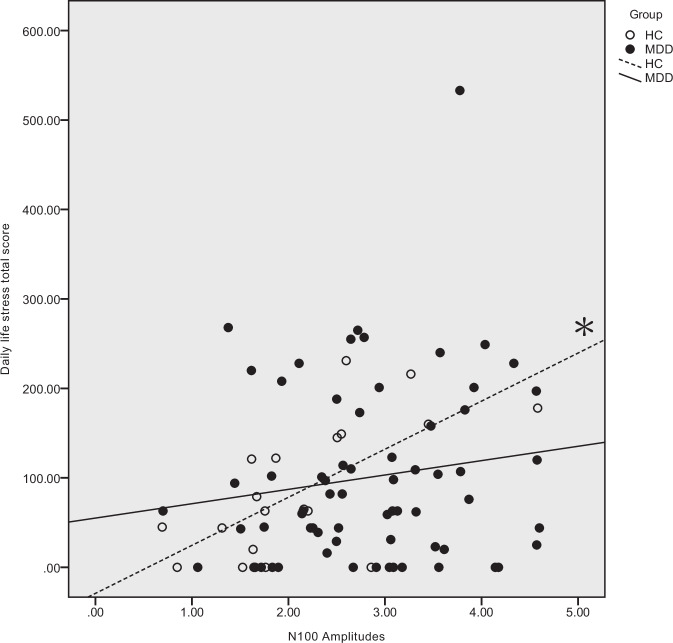
Association of N100 with daily life stress score in HC and MDD groups. A significant correlation of N100 over the left dorsolateral prefrontal cortex and daily life stress score is only found in the HC group. *p < 0.05.

Correlations between the left PFC N100 and clinical variables were analyzed separately for the HC and MDD groups. In the HC group, a significantly positive correlation was observed between N100 amplitude and the daily life stress total score (r = 0.653, p = 0.002), this relationship was not present in patients with MDD (Fig. 1). The N100 demonstrated significant inverse correlations with the CGI score (r = −0.249, p = 0.044) and the TRDSS (r = −0.243, p = 0.049) in patients with MDD.

**Fig. 2:**
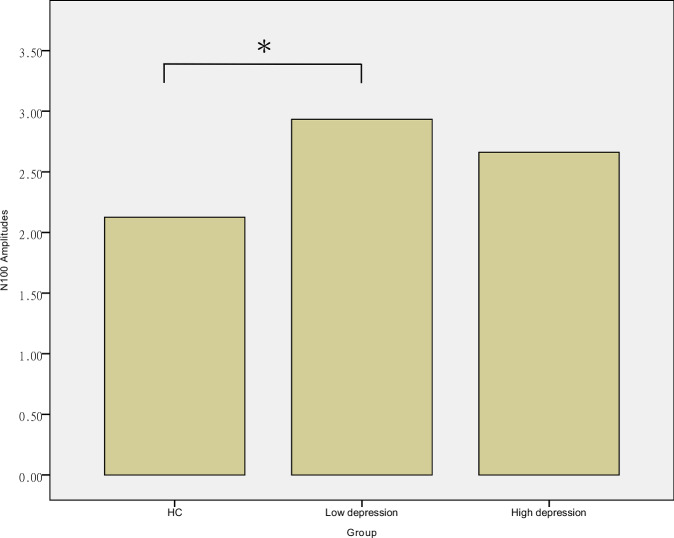
Comparison of N100 values (i.e., Y-axis) among healthy controls, low depression, and high depression groups. The highest N100 is found in the low depression group. *p < 0.05.

A significantly higher N100 value was observed in the MDD group compared to the HC group (p = 0.003). We also compared N100 amplitudes across the HC, low depression, and high depression groups. The main effect of group was statistically significant (F = 5.308, p = 0.007) and significant differences were found between the HC group and the low depression group (p = 0.002). However, no significant differences were found between the HC group and the high depression group (p = 0.062), nor between the low and high depression groups (p = 0.263) (Fig. 2). To further evaluate the relationship between treatment refractoriness and N100 amplitudes, a subgroup analysis was conducted comparing nTRD and TRD patients. No significant difference was found in N100 amplitudes between the nTRD and TRD groups. An ANOVA test of N100 amplitudes across the HC, nTRD, and TRD groups demonstrated a significant main effect of group (F = 5.020, p = 0.009) and significant differences were found between the HC and nTRD groups (p = 0.003), as well as between the HC and TRD groups (p = 0.014). Furthermore, a significant positive correlation was found between N100 amplitudes and the MSM score of treatment failures exclusively within the nTRD group (r = 0.388, p = 0.046). No significant correlations were found between N100 amplitudes and total MSM scores, MSM scores of symptom severity, MSM scores of treatment failure, or life stress scores in the nTRD group, the TRD group, or all MDD participants combined.

**Fig. 3:**
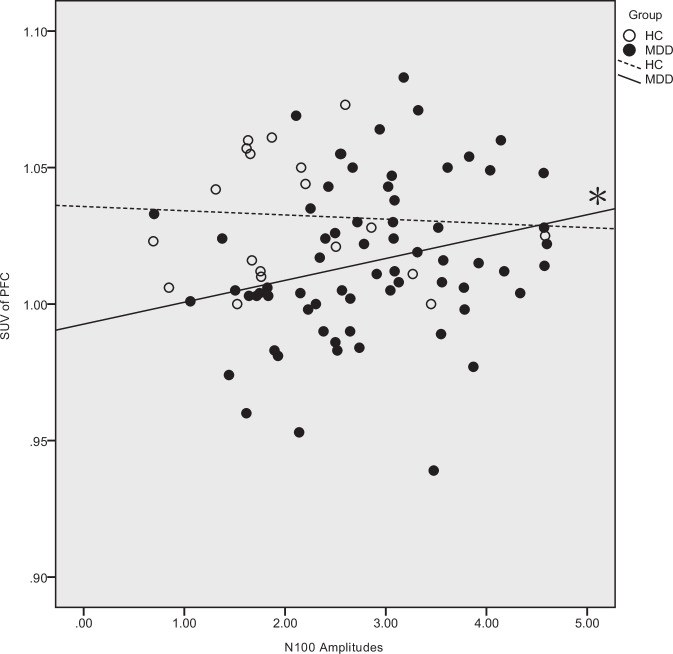
A significant correlation between SUV in the PFC and N100 only in the MDD group, not in the HC. *p < 0.05.

Significantly higher SUV levels were found in MDD patients compared to the HC (FWE-corrected p < 0.001) in regions including the bilateral striatum, amygdala, hippocampus, and temporal cortex. Voxel-wise analyses were conducted to examine associations between the N100 and regional brain glucose metabolism in both groups. In MDD patients, significantly positive correlations were noted over the superior frontal gyrus and left inferior frontal gyrus, while negative correlations were found in the right middle temporal cortex, right inferior parietal cortex, and middle cingulum (Table 3). In the HC group, a significantly positive correlation was found in the medial frontal gyrus, with negative correlations observed in the cerebellum and anterior cingulum. Moreover, SUV values from the prefrontal cortex (PFC) were extracted to analyze their connection with the N100. A significant correlation was found in the MDD group (r = 0.257, p = 0.037) but not in the HC group (Fig. 3).

**Table 3:** Correlations of prefrontal N100 and regional glucose metabolism in patients with MDD and healthy control (HC) subjects.

Brain Region	x,y,z (mm)	x,y,z (mm)	x,y,z (mm)	Cluster k	P(uncorrected)	T-value	Z-value	p(FDR-corrected)	p(SVC-corrected)
**MDD: Positive Correlations**									
Superior frontal gyrus (BA 10)	20	64	−14	1079	0.01618	3.955322	3.716878	0.614801	0.005**
Medial prefrontal cortex (BA10)	4	48	−9			3.035895	2.918181		
Medial prefrontal cortex (BA10)	14	46	8			2.658942	2.576771		
Left inferior frontal gyrus (BA 47)	−42	20	0	1175	0.01271	3.048718	2.929663	0.614801	0.028*
Left inferior frontal gyrus	−50	34	2			2.974271	2.862874		
Left middle frontal gyrus (BA 10)	−38	46	24			2.7852	2.691927		
**MDD: Negative Correlations**									
Right middle temporal cortex	48	−6	−20	2170	0.0014	4.444891	4.120492	0.04389*	
Right postcentral gyrus	52	−20	−16			4.089428	3.828983		
Right middle temporal cortex	50	−32	−18			3.802266	3.587527		
Right inferior parietal cortex (BA 40)	50	−44	54	2955	0.00031	4.091307	3.830546	0.02933*	
Right precentral gyrus (BA6)	38	2	28			3.772888	3.562529		
Right precentral gyrus	54	−10	42			3.332227	3.181152		
Middle cingulum (BA 24)	−4	−8	50	2301	0.00108	3.280232	3.135376	0.04389*	
Left inferior parietal cortex (BA 2)	−46	−27	46			2.848524	2.749386		
Left postcentral gyrus	−34	−12	34			2.830936	2.733448		
**HC: Positive Correlations**									
Medial frontal gyrus (BA11)	0	52	−12	653	0.02568	5.58349	4.045399	0.952513	0.003**
	10	52	−10			4.359757	3.45016		
	−14	46	−22			3.187001	2.741533		
HC: Negative Correlations									
Cerebellum	−8	−44	−50	1130	0.0051	4.933173	3.745125	0.334178	0.007**
Cerebellum	2	−48	−34			4.614839	3.58514		
Midbrain	6	−34	−16			3.399726	2.881642		
Anterior cingulum (BA 25)	4	11	−16	1226	0.0038	4.146745	3.332535	0.334178	0.036*

**p < 0.01.

*p < 0.05.

## Discussion

Our findings revealed that DLPFC N100 function was significantly higher in MDD groups than in the HC, as well as higher in low depression groups compared to the HC. In the HC group, there was a strong correlation between N100 value and the daily life stress score. In contrast, the N100 was significantly associated with the CGI and TRDSS score in MDD patients. The middle cingulum was negatively associated with N100 amplitude in MDD individuals, and a positive correlation with the prefrontal cortex was identified solely in the MDD group, but not in the HC.

The low depression group exhibited significantly greater frontal N100 amplitudes compared to the HC group. Growing evidence links GABA system dysfunction to MDD [28, 29], and prior TMS-EEG research indicates that N100 reflects MDD severity and antidepressant treatment response [30]. Moreover, the decrease in N100 following antidepressant treatment is considered evidence of restoration of the frontal excitatory/inhibitory balance, also known as the E/I balance [31]. While a negative correlation has been reported between changes in N100 amplitude and HDRS-17 scores in patients [15], no significant difference in N100 value was observed between the high and the low depression groups in our study. Taken together, these findings suggest that upregulated GABA-B receptor inhibition, indexed by the N100, is a prominent feature in low-severity and non-refractory depression. This is corroborated by our subgroup analysis, which revealed that while N100 amplitudes were significantly elevated in both nTRD and TRD groups relative to healthy controls, they correlated positively with the number of treatment failures exclusively within the nTRD group. This indicates that during the earlier, non-refractory stages of depression, the brain may actively upregulate GABA-B inhibition as a dynamic, compensatory response to accumulating treatment failures. However, as the disease progresses into full treatment resistance or high severity, this specific compensatory mechanism appears to reach a physiological ceiling and the linear relationship decouples. At these advanced stages, other distinct systems might predominate, such as the GABA-B receptor network related to long-interval cortical inhibition (LICI) [32, 33]. Because our study did not directly measure LICI or other cortical inhibition paradigms, this hypothesis remains speculative and requires validation through future multi-marker investigations.

The daily life stress score was found to be strongly associated with the N100 exclusively in HC individuals. Chronic stress may potentiate presynaptic GABA-B receptor function of corticotrophin-releasing hormone (CRH) neurons in the hypothalamic paraventricular nucleus (PVN) [34], and GABA-B receptor-mediated regulation is critical for stress resilience [35]. It is possible that while healthy subjects adaptively modulate the stress response through N100 regulation, pathological brain alterations in MDD patients may necessitate the compensatory recruitment of GABA-B receptor system for an adequate stress response.

A positive relationship between N100 amplitude and prefrontal SUV was observed exclusively in the MDD group. This finding can be understood through the lens of the excitatory/inhibitory (E/I) balance hypothesis. Magnetic resonance spectroscopy (MRS) literature indicates that MDD is characterized by a baseline GABAergic deficit and an overactive glutamatergic system in the prefrontal cortex, leading to excitotoxicity and hypermetabolism [29, 36, 37]. GABA-B receptors, which mediate the N100 potential, provide long-lasting inhibitory control. In this context, the elevated N100 amplitudes observed in our low-severity depression cohort likely reflect a dynamic, compensatory upregulation of GABA-B receptor sensitivity. Faced with an underlying GABAergic deficit and excessive excitatory drive (evidenced by increased prefrontal SUV), the cortical network actively enhances GABA-B-mediated inhibition to restore E/I balance. However, as depression severity progresses, this compensatory mechanism may become exhausted or insufficient, highlighting the N100 as a state-dependent marker of network distress.

In voxel-wise analysis, the right middle temporal cortex, right inferior parietal cortex, and middle cingulate cortex all revealed a negative correlation with N100 amplitude in MDD patients. Previous research found that alterations in the temporal-parietal-limbic network, particularly changes in gray matter volumes of the right middle temporal gyrus, right parietal lobe, and the cingulated gyrus, were present in MDD subjects with a history of suicidality, correlating with the scores of dysfunctional attitude scales [38, 39]. In MDD patients with a history of suicide attempts, smaller hippocampus, which is a component of the medial temporal lobe, was reported to predict acute suicide attempts [40]. Additionally, a reduction in magnetization transfer ratio, which is indicative of microstructural tissue damage, has been observed in the bilateral parietal lobules [41]. Notably, recent findings demonstrate potentiated N100 amplitudes in patients with suicidal ideation relative to non-suicidal patients and the HC [42]. Collectively, N100 amplitudes may be associated with the neural circuitry underlying suicidality and, therefore, may serve as a potential biomarker for suicide risk, though more research is needed.

Regarding demographic variables, the higher proportion of females in our MDD cohort aligns with the widely recognized epidemiological sex differences in depression prevalence. Importantly, our analysis revealed no significant gender-related effects on N100 amplitudes across the whole study population or within the individual groups. The lack of sex differences in our neurophysiological data may be partially attributed to our methodology, wherein TMS stimulation intensity was individualized to 120% of each participant’s resting motor threshold, a standard protocol that helps control for baseline anatomical variations that can differ between genders.

The first limitation of this study is its narrow electrophysiological focus on the GABA-B receptor-related N100. Because we did not concurrently measure other TMS-EEG markers of cortical inhibition or incorporate markers for dopaminergic and glutamatergic pathways, we lack direct evidence to fully characterize the complex, shifting neural mechanisms that may predominate at higher levels of depression severity. Additionally, although our study utilizes 18F-FDG PET to reflect general downstream cerebral glucose metabolism, this imaging modality does not directly quantify specific neurotransmitter receptor density. Given that the N100 is fundamentally mediated by GABA-B receptors, a notable limitation is the absence of specific molecular imaging. Future studies combining TMS-EEG with GABA-receptor-specific PET radioligands would be highly valuable. Such multi-modal investigations could determine whether the altered N100 amplitudes observed in MDD are driven by underlying changes in GABA-B receptor density and availability, or by altered binding affinity, thereby offering a direct molecular-to-electrophysiological bridge to fully characterize fronto-limbic E/I imbalances. Nonetheless, when considered alongside previous research, the significance and function of N100 in MDD patients remain clear. Second, the cross-sectional design of this article limited the ability to determine causal relationships. Future longitudinal studies are needed to better understand the link between depression severity, N100 abnormalities, and changes in brain metabolism. Moreover, even though our analysis indicated that the specific class of antidepressant monotherapy did not significantly alter N100 amplitudes, our dataset lacked specific daily dosages and treatment durations, preventing the evaluation of potential cumulative or dose-dependent effects on cortical inhibition. Finally, while our study includes an electrophysiological and clinical subgroup analysis between TRD and non-TRD patients, a detailed comparison of cerebral glucose metabolism between these specific groups was not conducted. Since the N100 has been linked to treatment response and symptom remission [14, 30], it is plausible that distinct connections exist between brain metabolism and TMS-EEG markers when comparing these two groups. Future multimodal studies should specifically investigate whether the relationship between cortical inhibition and regional brain metabolism differs between refractory and non-refractory states.

In conclusion, our study demonstrates that left prefrontal N100 amplitudes are significantly elevated in patients with low-severity and non-treatment-resistant depression (nTRD) compared to healthy controls. Furthermore, the N100 exhibits a distinct positive correlation with prefrontal glucose metabolism exclusively in the MDD cohort, while negatively associating with metabolism in the temporal-parietal-limbic network. These findings suggest that upregulated GABA-B receptor-mediated inhibition serves as a dynamic, compensatory neurophysiological response aiming to restore the excitatory/inhibitory (E/I) balance in the face of aberrant fronto-limbic metabolism. Clinically, the N100 waveform emerges as a promising, state-dependent biomarker for evaluating depression severity, network distress, and potential suicide risk. Future research should employ longitudinal designs and multi-modal approaches combining TMS-EEG with GABA-specific PET radiotracers to definitively link functional electrophysiology with molecular receptor availability, and to further differentiate the neurophysiological mechanisms governing refractory versus non-refractory depressive states.

## Data Availability

The datasets generated and analyzed during the current study are available from the corresponding author upon reasonable request.

## Code availability

The custom MATLAB scripts used for EEG analysis in this study are available from the corresponding author upon reasonable request.
